# Lofgren Syndrome: Achieving an Accurate Diagnosis for Improved Patient Care

**DOI:** 10.7759/cureus.51801

**Published:** 2024-01-07

**Authors:** Leonor Gama, Ana Santos e Silva, Ana Valido, Josiana Duarte, Henrique Rita

**Affiliations:** 1 Internal Medicine, Hospital do Litoral Alentejano, Santiago do Cacém, PRT; 2 Internal Medicine, Unidade Local de Saúde do Litoral Alentejano, Santiago do Cacém, PRT; 3 Rheumatology, Hospital do Litoral Alentejano, Santiago do Cacém, PRT

**Keywords:** subcutaneous nodules, bimalleolar edema, differential diagnosis, sarcoidosis, lofgren syndrome

## Abstract

Lofgren syndrome is a clinically distinct phenotype of sarcoidosis. It is characterized by the triad of bilateral hilar lymphadenopathy, arthritis (usually the ankles), and fever. We present the case of a 31-year-old male patient who presented with fever and edema in both lower limbs, with palpation of subcutaneous nodules. A chest contrast-enhanced computerized axial tomography (CECT) scan revealed perihilar and mediastinal lymphadenopathy. In making the diagnosis, tuberculosis and lymphoma were both ruled out. A mediastinoscopy confirmed Lofgren syndrome. In medicine, a good differential diagnosis is important, as it will help inform the best treatment for the patient.

## Introduction

Lofgren syndrome was first described in 1952 and is characterized by the triad of bilateral hilar lymphadenopathy, arthritis (usually of the ankles), and fever. It generally presents at the ages of 25 and 40, with a second peak at ages 45 and 60, and is more common in females [1]. Despite presenting with granulomas, it is a clinically distinct phenotype from sarcoidosis [2]. The onset is acute, unlike sarcoidosis, which is more insidious and has slower progression. Ninety-five percent of cases with this condition present benign pathology without sequelae and without treatment, although some cases may require immunosuppression to achieve complete remission [3,4]. The diagnosis is based on clinical, imaging data, and histological findings, with the already specified triad having high sensitivity and specificity [5].

## Case presentation

We present a male patient, 31 years old, white, born and residing in Portugal, working in a car parts sales factory. The patient is a smoker with an estimated smoking history of 13 pack years. He denied using chronic medication. He was electively admitted to an internal medicine service to study bimalleolar edema with four weeks of evolution. He reported having a fever (mainly at night, maximum temperature measured at 39ºC in the axilla) and night sweats. A physical examination highlighted edema in both lower limbs, with palpation of subcutaneous nodules.

The blood test analysis evidenced high inflammatory parameters, namely, a C-reactive protein (CRP) of 11 mg/dl and a sedimentation rate of 75 mm/h. From the urinary study, the absence of proteinuria stood out, which ruled out nephrotic syndrome. Of the remaining studies, HIV, hepatitis B and C, and syphilis serology were negative, autoimmunity and angiotensin-converting enzyme (ACE) were unchanged, and interferon-gamma release assay (IGRA) was negative.

**Figure 1:**
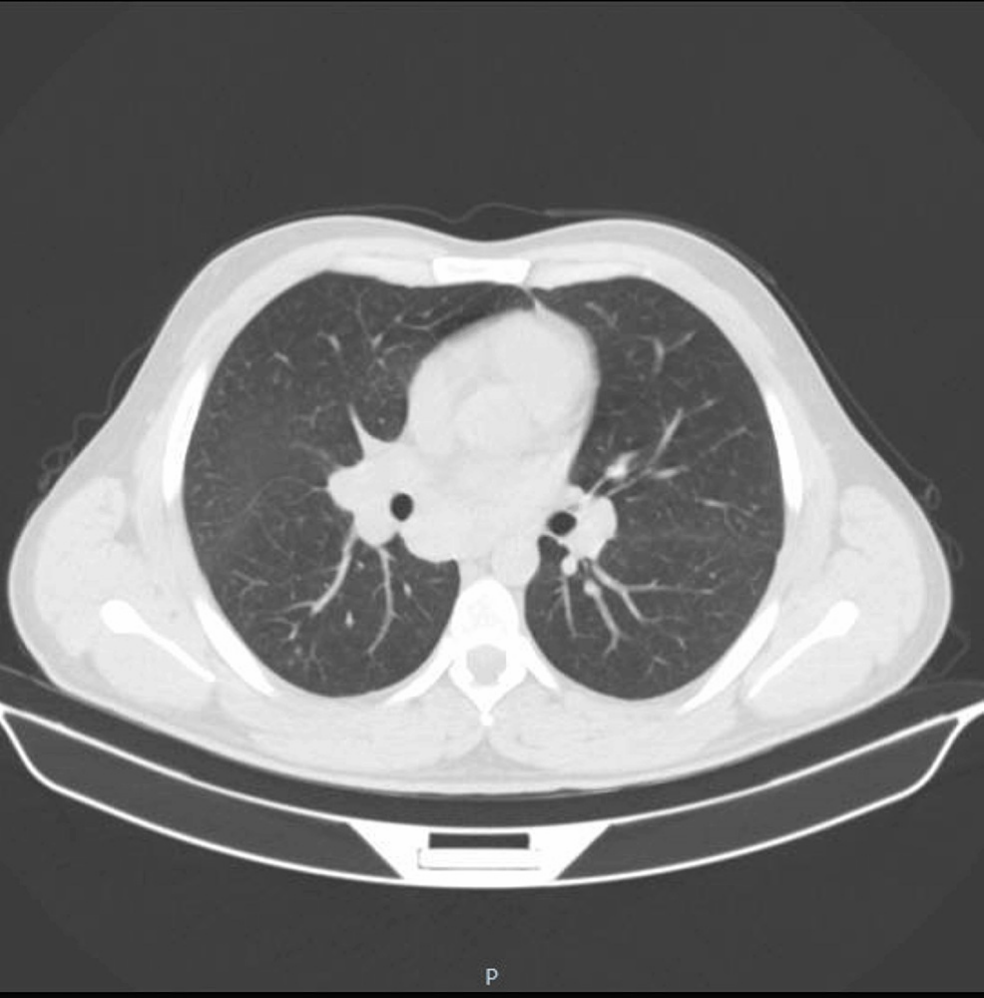
CT scan showing perihilar lymphadenopathy

**Figure 2:**
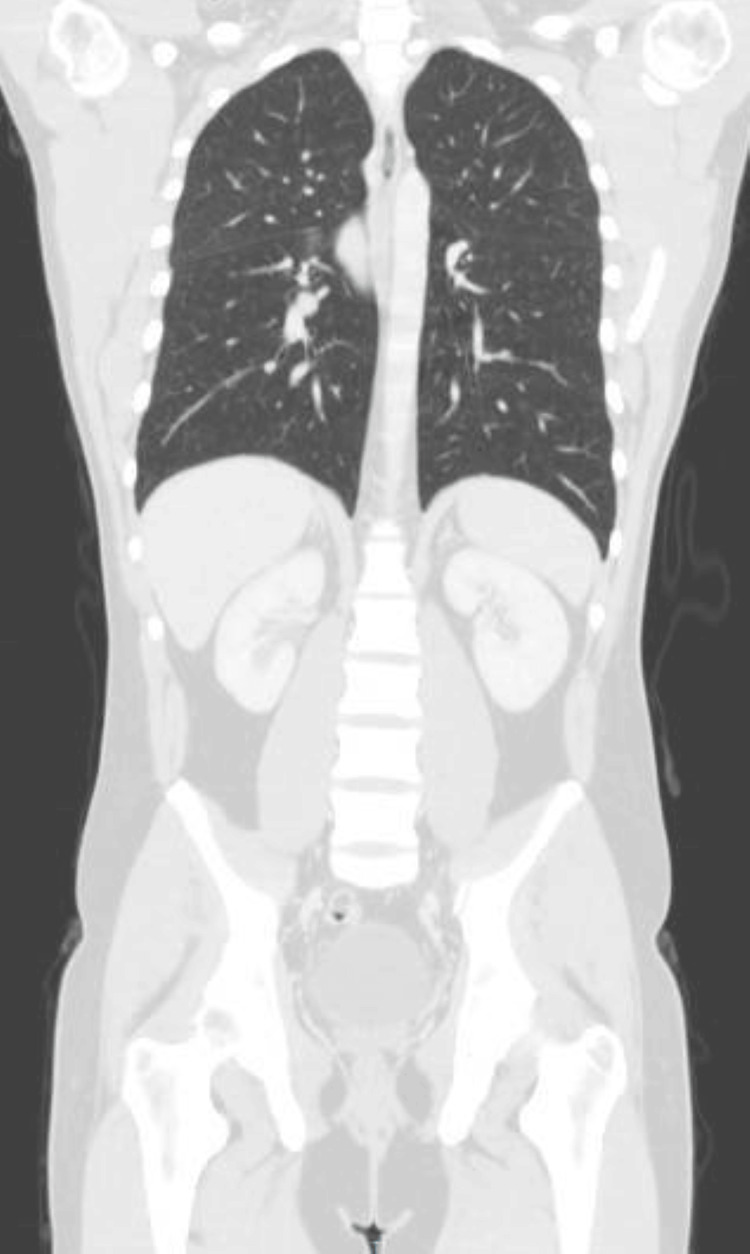
CT scan showing perihilar and mediastinal lymphadenopathy

On the chest X-ray, the patient showed perihilar reinforcement, mainly in the right hilum, which is why a contrast-enhanced computerized axial tomography (CECT) scan was then carried out (Figures 1, 2), which revealed perihilar and mediastinal lymphadenopathy, with no parenchymal involvement.

**Figure 3:**
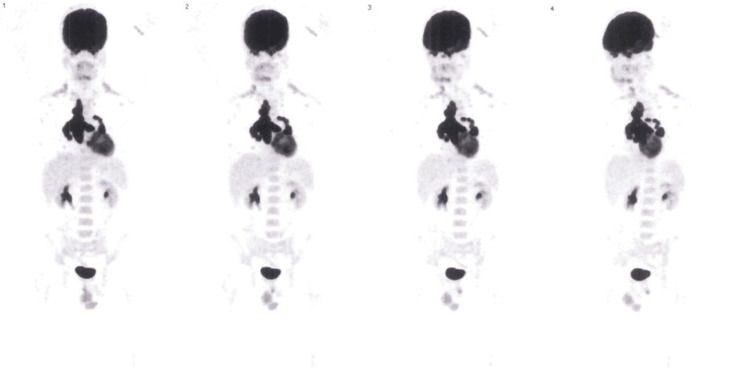
PET demonstrates anomalous hypermetabolism in bilateral perihilar and mediastinal lymph nodes PET: positron emission tomography

As it was imperative to exclude lymphoproliferative disease, positron emission tomography (PET) was performed (Figure 3), which revealed anomalous hypermetabolism in the bilateral perihilar pulmonary and mediastinal lymph nodes.

Therefore, a mediastinoscopy was performed, which showed, surprisingly, that the pathological anatomy demonstrated non-necrotizing granulomas, with a negative mycobacteria (*Mycobacterium tuberculosis)* CB-NAAT (cartridge-based nucleic acid amplification test).

During the diagnostic process, the symptoms spontaneously reversed without medication; therefore, the diagnosis of Lofgren syndrome was made. The patient is currently undergoing follow-up consultations in rheumatology, internal medicine, and pulmonology, and he is asymptomatic.

## Discussion

Sarcoidosis, particularly Lofgren syndrome, can encompass numerous different clinical presentations [6]. The clinical course of sarcoidosis is variable and sometimes unpredictable, ranging from acute and self-limited to chronic, progressive, and debilitating, whereas, with Lofgren syndrome, the symptoms usually reverse without medication [3]. The most commonly affected organs are the lungs, although a proportion of patients show extrapulmonary involvement such as the skin, lymph nodes, and eyes [3]. A diagnosis of Lofgren syndrome is accepted when compatible clinical and radiological findings are present. However, it is important to rule out primary pulmonary tuberculosis [1]. Malignancies, specifically lymphoma, must also be excluded because of their high prevalence in young people.

This case study highlights the importance of a good clinical history and why it is imperative to put forward all diagnostic hypotheses to obtain a correct diagnosis for the patient.

The beauty of internal medicine is that it is based on the principle that each patient should be treated holistically so that an accurate diagnosis can be made by considering all possible outcomes.

## Conclusions

Lofgren syndrome is an acute presentation of sarcoidosis. The main differential diagnosis includes infections, especially tuberculosis, and malignancies, in particular lymphoma. In this case, everything seemed to be in line with a proliferative disease, but the biopsy was surprising. In medicine, a good differential diagnosis is important in order to provide patients with the correct care.
